# Evidence for Mitochondrial Respiratory Deficiency in Rat Rhabdomyosarcoma Cells

**DOI:** 10.1371/journal.pone.0008637

**Published:** 2010-01-08

**Authors:** Vanessa E. Jahnke, Odile Sabido, Aurélia Defour, Josiane Castells, Etienne Lefai, Damien Roussel, Damien Freyssenet

**Affiliations:** 1 Université de Lyon, Université Jean Monnet, Laboratoire de Physiologie de l'Exercice EA4338, Saint Etienne, France; 2 Université de Lyon, Université Jean Monnet, Centre Commun de Cytométrie en Flux, Saint Etienne, France; 3 Université de Lyon, Université Claude Bernard Lyon 1, Régulations Métaboliques Nutrition et Diabètes INSERM U870, Oullins, France; 4 Université de Lyon, Université Claude Bernard Lyon 1, Laboratoire de Physiologie Intégrative Cellulaire et Moléculaire CNRS U5123, Villeurbanne, France; Universidad Europea de Madrid, Spain

## Abstract

**Background:**

Mitochondria can sense signals linked to variations in energy demand to regulate nuclear gene expression. This retrograde signaling pathway is presumed to be involved in the regulation of myoblast proliferation and differentiation. Rhabdomyosarcoma cells are characterized by their failure to both irreversibly exit the cell cycle and complete myogenic differentiation. However, it is currently unknown whether mitochondria are involved in the failure of rhabdomyosarcoma cells to differentiate.

**Methodology/Principal Findings:**

Mitochondrial biogenesis and metabolism were studied in rat L6E9 myoblasts and R1H rhabdomyosacoma cells during the cell cycle and after 36 hours of differentiation. Using a combination of flow cytometry, polarographic and molecular analyses, we evidenced a marked decrease in the cardiolipin content of R1H cells cultured in growth and differentiation media, together with a significant increase in the content of mitochondrial biogenesis factors and mitochondrial respiratory chain proteins. Altogether, these data indicate that the mitochondrial inner membrane composition and the overall process of mitochondrial biogenesis are markedly altered in R1H cells. Importantly, the dysregulation of protein-to-cardiolipin ratio was associated with major deficiencies in both basal and maximal mitochondrial respiration rates. This deficiency in mitochondrial respiration probably contributes to the inability of R1H cells to decrease mitochondrial H_2_O_2_ level at the onset of differentiation.

**Conclusion/Significance:**

A defect in the regulation of mitochondrial biogenesis and mitochondrial metabolism may thus be an epigenetic mechanism that may contribute to the tumoral behavior of R1H cells. Our data underline the importance of mitochondria in the regulation of myogenic differentiation.

## Introduction

Adult skeletal muscle fibers are formed via the fusion of individual myoblasts during development. Although multinucleated muscle fibers are considered to be permanently differentiated and therefore incapable of mitotic activity, skeletal muscle retains the capacity to repair and regenerate, mainly due to the presence of satellite cells. During muscle regeneration after injury, satellite cells are activated, proliferate by multiple rounds of cell division, fuse together and with existing damaged muscle fibers to form differentiated muscle fibers. Myoblats can also return to quiescence and contribute to the self-renewal of satellite cell population (reviewed in [1]). Recapitulation of the myogenic program requires energy production for the execution of a number of regulatory biosynthesis events such as DNA synthesis, mitosis but also protein and lipid synthesis. As the major energy source in most of cells, mitochondrial oxidative phosphorylation may therefore play important regulatory roles during myogenesis.

Rhabdomyosarcoma, the most common soft tissue sarcoma in children and adolescents, arises from immature cells that are destined to form striated skeletal muscle [2]. Although rhabdomyosarcoma cells express a number of myogenic-dependent proteins [3], [4], [5], these cells are characterized by their failure to both irreversibly exit the cell cycle and complete skeletal muscle differentiation program [4]. Therefore, rhabdomyosarcoma cells constitute an interesting model to study the mechanisms that control myogenic differentiation.

The incapacity of rhabdomyosarcoma cells to differentiate has been assigned to different chromosomal abnormalities. For example, two chromosomal translocations, t(2;13)(q35;q14) and t(1;13)(p36;q14), are associated with numerous alveolar rhabdomyosarcomas, the resulting fusion proteins (PAX3-FKHR and PAX7-FKHR) acquiring the capacity to inhibit MyoD and the subsequent differentiation of rhabdomyosarcoma cells [6], [7], [8]. Besides the genetic characterization of rhabdomyosarcoma, epigenetic mechanisms may also contribute to the failure of rhabdomyosarcomas cells to differentiate. In 1956, Otto Warburg described that experimentally-induced tumour cells exhibited a reduced oxidative phosphorylation and an increased glycolysis [9], raising the possibility that the original non-carcinogenic phenotype of these mammalian cells was regulated by mitochondrial oxidative phosphorylation. This hypothesis is supported by a number of studies showing that experimental inhibition or activation of mitochondrial biogenesis and mitochondrial metabolism strongly modulates *in vitro* and *in vivo* oncogenic phenotype [10], [11], [12], [13], [14], [15]. Importantly, this regulatory function of mitochondria seems to be particularly relevant for the regulation of myogenic differentiation. *In vivo* and *in vitro* myogenesis is accompanied by a tight regulation of mitochondrial biogenesis [16], [17], [18]. Furthermore, the disruption of mitochondrial membrane potential in C2C12 myoblasts is responsible for an invasive behaviour [10], [19]. Conversely, the stimulation of mitochondrial oxidative metabolism by pyruvate blocks the proliferation of L6E9 muscle cells [20], and the stimulation of mitochondrial biogenesis induces the expression of myogenin and muscle-specific genes [21], [22]. A defect in mitochondrial biogenesis and/or mitochondrial metabolism may thus contribute to the failure of rhabdomyosarcoma cells to exit the cell cycle and differentiate.

Here, we report that the expression of mitochondrial proteins and mitochondrial biogenesis factors is markedly increased in rhabdomyosarcoma cells, together with a large decrease in cardiolipin content. The differential regulation of mitochondrial protein expression and cardiolipin content was associated with major deficiencies in both basal and maximal mitochondrial respiration rates. These findings indicate that a defect in the regulation of mitochondrial biogenesis and mitochondrial metabolism is an epigenetic mechanism that may contribute to the tumoral behavior of R1H cells. Furthermore, they establish functional links between the regulation of mitochondrial biogenesis and the regulation of myogenic differentiation.

## Results

### Cell Cycle Analysis of L6E9 and R1H Rhabdomyosarcoma Cells

**Figure 1:**
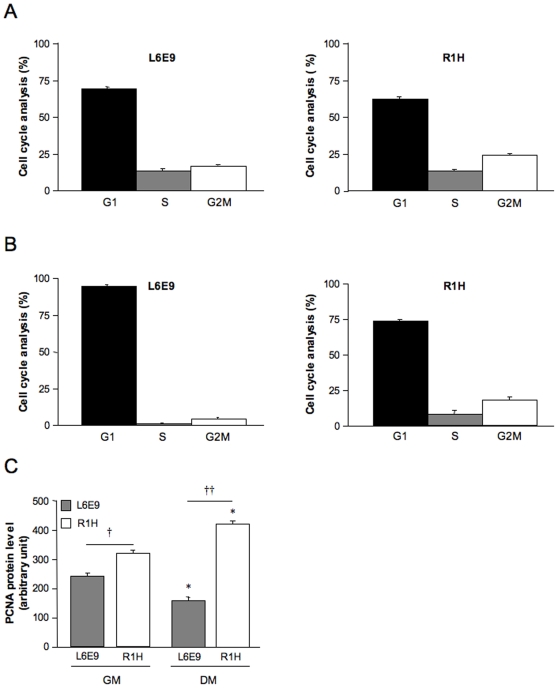
Cell cycle analysis and proliferating cell nuclear antigen (PCNA) protein content in L6E9 and R1H cells. Cell cycle analysis of L6E9 myoblasts and R1H cells cultured in growth medium (A) and differentiation medium for 36 hours (B). Cell cycle analysis was performed after doublet exclusion on morphologically normal living cells. (C) PCNA protein level in L6E9 myoblasts and R1H cells cultured in growth medium (GM) and differentiation medium (DM). PCNA immunolabeling was performed after doublet exclusion on morphologically normal fixed cells. Data are means ± SE from 6 culture dishes. * *P*<0.05: significantly different from corresponding cells in GM; † *P*<0.05 and †† *P*<0.01: significantly different from L6E9 myoblasts.

Figure 1 indicates the relative distribution of L6E9 and R1H cells in G1, S and G2M phases when cultured with growth medium (Figure 1A) and differentiation medium (Figure 1B). In agreement with the tumoral behavior of rhabdomyosarcoma cells, R1H cells cultured with differentiation medium for 36 hours failed to exit the cell cycle and were still cycling, as evidenced by the presence of cells in S (11.6%) and G2M (20.8%) phases. For comparison purpose, 0.5% and 8.9% of L6E9 cells were in S and G2M phases, respectively. In agreement with these observations, PCNA, a cofactor of DNA polymerase δ during S phase, whose expression was reduced by 50% at the onset of differentiation in L6E9 cells, was even significantly increased in R1H cells cultured with differentiation medium, further illustrating the failure of R1H cells to exit the cell cycle (Figure 1C).

### Regulation of Mitochondrial Biogenesis

**Figure 2:**
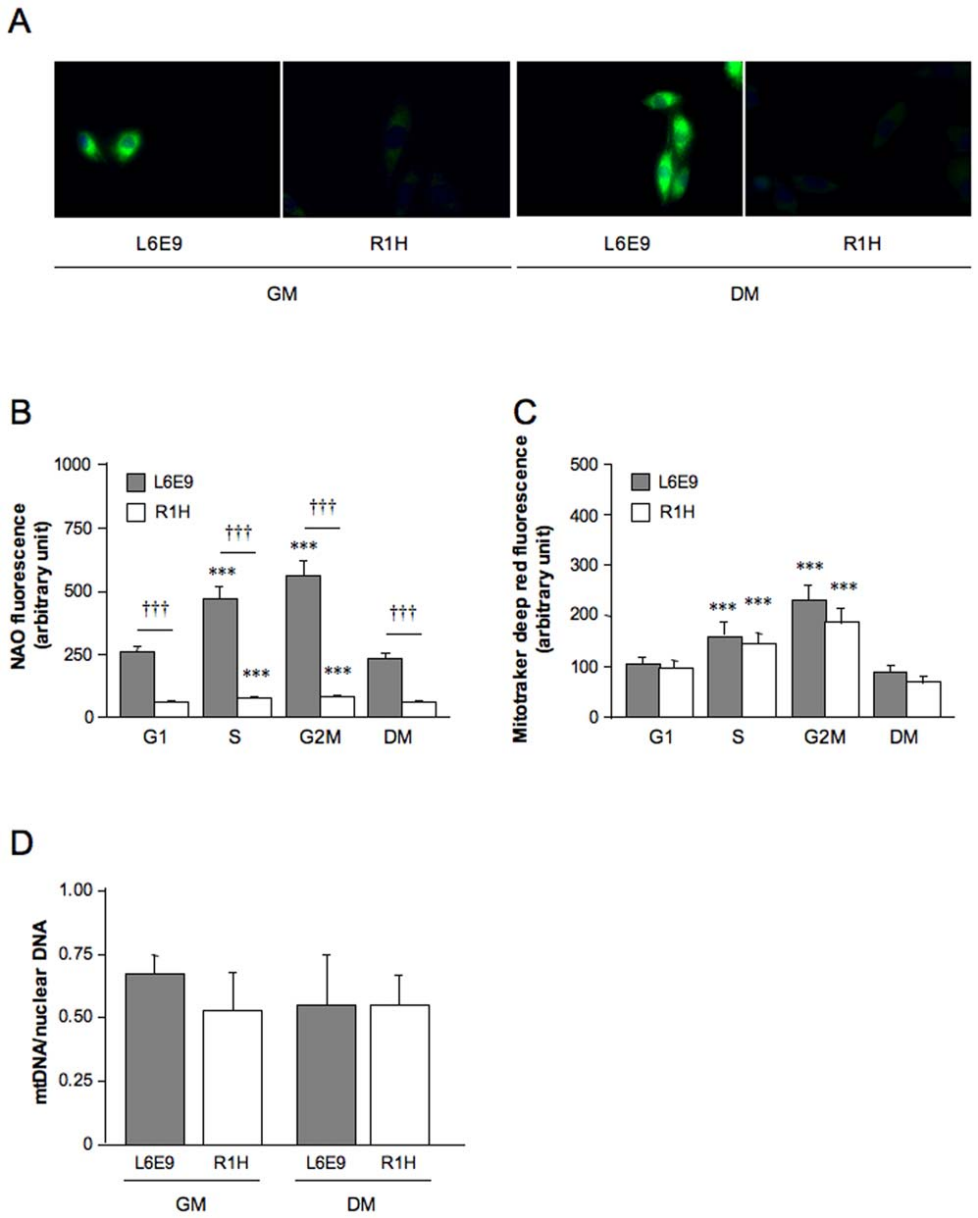
Cardiolipin content, mitochondrial content and mtDNA content. (A) Representative image of mitochondrial staining by nonyl acridine orange (NAO) in L6E9 myoblasts and R1H cells cultured in growth medium (GM) and in differentiation medium (DM) for 36 hours. Nuclei were stained with Hoechst 33342. Cells were visualized by fluorescence microscopy (Olympus inverse microscope IX81 system). (B) Cardiolipin content was determined from the geometric means of height NAO fluorescence signal. (C) Mitochondrial content was assessed from the geometric means of Mitotracker deep red fluorescence signal. Flow cytometry analysis was performed on living cells after doublet exclusion as a function of L6E9 and R1H cells position in the cell cycle (G1, S and G2M). Cells were cultured in GM and DM. (D) mtDNA quantity calculated as the ratio of mitochondrial subunit II of cytochrome oxidase to peptidylprolyl isomerase A DNA levels determined by real-time PCR in L6E9 and R1H cultured in GM and DM. Data are means ± SE from 6 culture dishes. *** *P*<0.001: significantly different from corresponding cells in G1 phase; ††† *P*<0.001: significantly different from L6E9 myoblasts.

We first determined the content of cardiolipin, the phospholipid signature of the mitochondrial inner membrane that accounts for 20% of mitochondrial phospholipids [23]. When observed under fluorescence microscopy, L6E9 mitochondria stained with NAO, a fluorophore which binds to cardiolipin [24], appeared bright, which contrasted with the faint intensity of NAO staining in R1H cells (Figure 2A). Quantitative analysis of NAO fluorescence by flow cytometry corroborated this observation (Figure 2B). Furthermore, R1H cells poorly increased their cardiolipin content during the cell cycle (37%), whereas a two-fold increase was observed in L6E9 cells. To quantitate directly the regulation of mitochondrial content, we next used a fluorochrome (MitoTracker Deep Red) that yields a fluorescence signal proportional to the density of mitochondria. As shown in Figure 2C, the mitochondrial content was increased by about 2-fold in G2M phases compared to G1 phases in L6E9 and R1H cells. In agreement with these observations, the ratio of mitochondrial DNA to nuclear DNA was similar in L6E9 and R1H cells (Figure 2D). Overall, these data suggest that a progressive depletion of the cardiolipin content occurs as R1H cells divide, while mitochondrial content is preserved.

We next investigated the expression of mitochondrial biogenesis factors and respiratory chain proteins (Table 1). As previously observed [25], expression of mitochondrial proteins and mitochondrial biogenesis factors in L6E9 myoblasts was decreased at the onset of differentiation. One reminiscent feature of this analysis was the higher amount of mitochondrial respiratory chain proteins in R1H cells when compared to L6E9 cells. This pattern was particularly marked for cells cultured in differentiation medium. In agreement with these data, the protein content of mitochondrial biogenesis factors was also higher in R1H cells compared to L6E9 cells. Most importantly, the decrease in the expression of mitochondrial biogenesis factors and respiratory chain proteins observed in L6E9 cells cultured with differentiation medium for 36 hours did not occur in R1H cells. Altogether these data indicate that the mitochondrial inner membrane composition and the overall process of mitochondrial biogenesis are markedly altered in R1H cells.

**Table 1:** Protein content of mitochondrial proteins and mitochondrial biogenesis factors.

	Growth medium		Differentiation medium	
	L6E9	R1H	L6E9	R1H
Core 2	101.2±6.0	228.1±21.2†††	67.5±3.4***	460.6±53.8**, †††
13.4	130.8±7.0	462.0±34.7†††	66.6±4.3***	456.6±19†††
20D6	93.4±3.1	121.8±19.5	76.3±4.9*	166.8±27††
PGC-1α	87.9±4.5	117.4±9.0†	9.2±0.6**	120.0±2.7†††
PPARα	74.8±4.3	58.1±14.4†	5.7±0.3***	95.4±4.2**, †††
PPARδ	85.6±3.5	128.0±23.0	12.2±0.7***	125.5±10.4†
NOS1	56.8±2.6	81.7±8.9†	5.5±0.4***	105.3±8.3†††

Analyses were performed on cells cultured in growth medium and differentiation medium. Geometric means of the fluorescence intensity peaks were used to determine the protein content. Data are means ± SE from 6 culture dishes. * *P*<0.05, ** *P*<0.01 and *** *P*<0.001: significantly different from corresponding cells in GM; † *P*<0.05, †† *P*<0.01 and ††† *P*<0.001: significantly different from L6E9 cells.

### Cellular Metabolism

**Figure 3:**
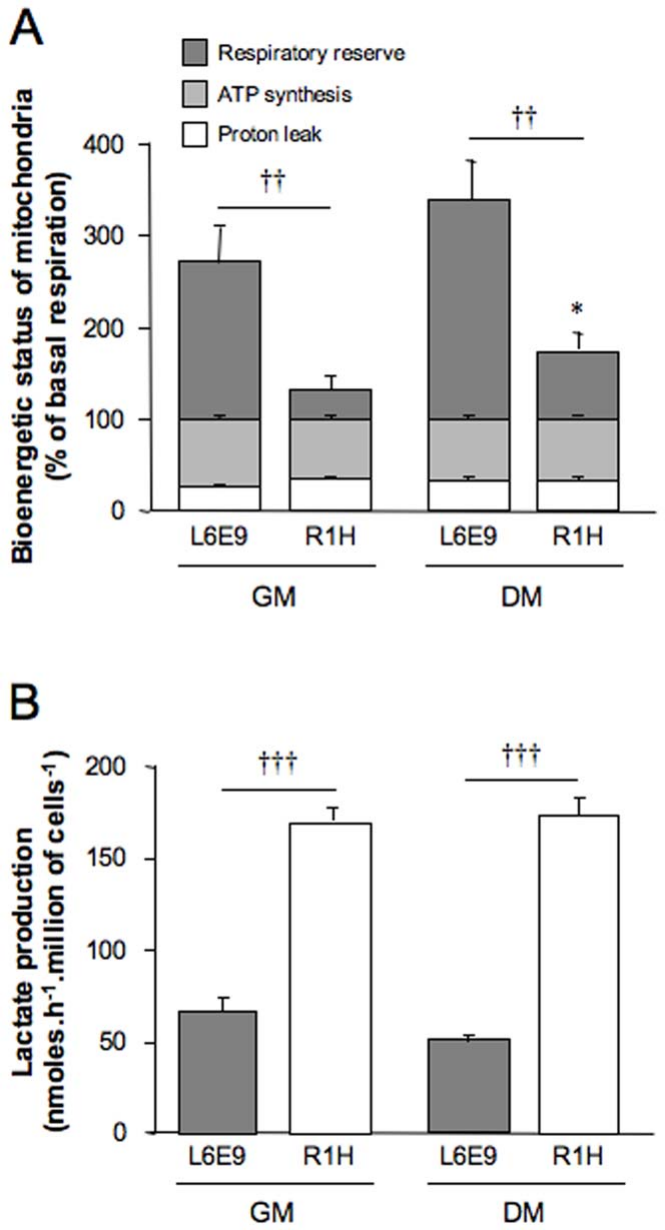
Bioenergetic status of L6E9 and R1H cells. (A) Mitochondrial proton leak (oligomycin insensitive respiration), mitochondrial ATP synthesis (basal respiration minus oligomycin-insensitive respiration) and mitochondrial respiratory reserve (FCCP-stimulated respiration minus basal respiration) are expressed as a percent of corresponding basal mitochondrial respiration reported in Table 2. Data are means ± SE from 12 independent culture dishes cultured in growth medium (GM) and differentiation medium (DM). (B) Lactate production of L6E9 and R1H cells cultured in GM and DM. Data are means ± SE from 6 independent culture dishes. *P<0.05: significantly different from corresponding cells in GM; †† *P*<0.01: significantly different from L6E9 myoblasts.

To test the bioenergetic relevance of these observations, mitochondrial respiration was determined. The rate of basal respiration was dramatically lower in R1H cells than in L6E9 cells both in growth and differentiation media (Table 2). Similarly, oligomycin-insensitive respiration was lower in R1H cells compared to L6E9 cells. The rate of FCCP-stimulated maximal respiration was even more markedly reduced. When expressed as a percent of basal respiration, oxygen consumption dedicated to mitochondrial ATP synthesis accounted for about 70%, whereas proton leak across the mitochondrial inner accounted for the remaining 30% (Figure 3A). This was consistently observed in L6E9 and R1H cells both in growth medium and differentiation medium. Despite lower rates of oxygen consumption, mitochondria of R1H cells thus allocated the same proportion of energy to produce ATP or to counteract proton leakage when compared to L6E9 cells, indicating that mitochondrial inner membrane permeability was similar between L6E9 and R1H cells. By contrast, the mitochondrial respiratory reserve (FCCP-induced maximal respiration rate minus basal endogenous respiration rate) was dramatically reduced in R1H cells. Therefore, the endogenous respiration of R1H cells was almost maximally stimulated in the basal state, with mitochondria operating at 80% and 60% of maximal respiration under growth and differentiation conditions, respectively. For comparison purposes, L6E9 cells retained a large mitochondrial respiratory reserve, leading mitochondria to operate at 37% and 30% of maximal respiration under growth and differentiation conditions, respectively. Reduced mitochondrial respiration suggests that R1H cells should have switch to a glycolytic energy production to sustain the rate of proliferation. Accordingly, lactate production by R1H cells was about 3-fold higher than the one reported for L6E9 cells (Figure 3B).

**Table 2:** Oxygen consumption rates of L6E9 and R1H cells.

	Growth medium		Differentiation medium	
	L6E9	R1H	L6E9	R1H
Basal	33.6±3.7	13.8±1.2†††	27.5±1.6	18.2±1.0*, †††
Oligomycin-insensitive	8.7±1.0	4.7±0.8††	9.2±1.6	5.9±1.4
FCCP-stimulated	91.1±16.6	18.2±3.0†††	93.0±14.3	31.7±3.7*, ††
RCR	10.4±3.0	3.9±0.9†	10.1±3.4	5.4±1.0

Oxygen consumption rates were measured in L6E9 myoblasts and R1H cells culture in growth medium or in differentiation medium at 37°C by polarography in DMEM serum free (basal), in the presence of oligomycin, and after the addition of FCCP. Respiration rates are expressed in nmol O_2_.min^−1^.cell^−1^. Respiratory control ratio (RCR) was calculated as the ratio between FCCP-stimulated and oligomycin insensitive respiration rates. For more details see Materials and Methods. Data are means ± SE from 12 independent culture dishes. ^*^ *P*<0.05, significantly different from corresponding cells in GM. † *P*<0.05, †† *P*<0.01 and ††† *P*<0.001: significantly different from L6E9 cells.

### Limited Import of Mitochondrial Protein Does Not Account for the Decrease in Mitochondrial Respiration in R1H Cells

**Figure 4:**
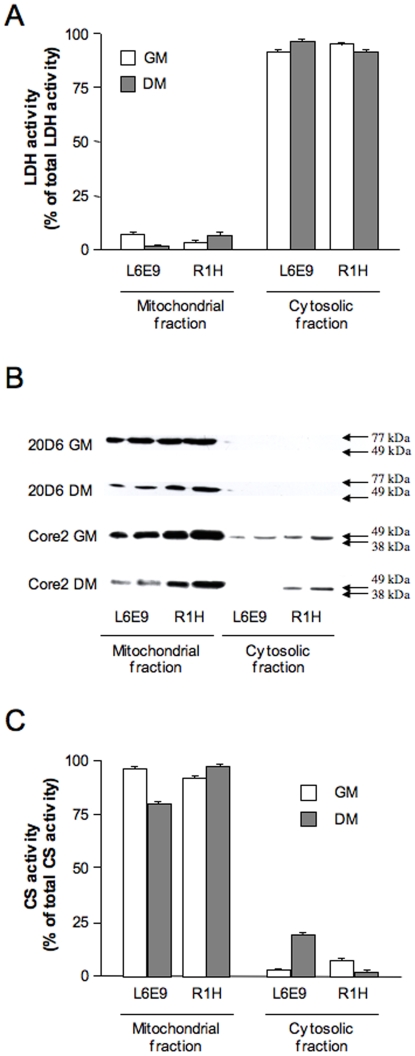
Mitochondrial import of Core 2 subunit of complex III, ATP synthase α (20D6) and citrate synthase. Lactate dehydrogenase (LDH) activity (A), Core 2 subunit of complex III and 20D6 protein content (B) and citrate synthase (CS) activity (C) were determined in mitochondrial and cytosolic fractions of L6E9 and R1H cells cultured in growth medium (GM) and differentiation medium (DM) for 36 hours. Data are means ± SE from 3 culture flasks.

With the exception of 13 proteins encoded by the mitochondrial genome, all others mitochondrial proteins are encoded by the nuclear genome and thus need to be imported in mitochondria [26]. A defect in mitochondrial import leading to a cytosolic accumulation of mitochondrial proteins could therefore explain the paradoxical observation that mitochondrial protein content was increased in R1H cells, together with a marked decrease in mitochondrial respiration. To examine this hypothesis, cytosolic and mitochondrial fractions were prepared and analyzed for the expression of Core 2 subunit of complex III and ATP synthase α protein content. Purity of the mitochondrial fraction was clearly indicated by the level of lactate dehydrogenase activity, which represented less than 5% of total lactate dehydrogenase activity (Figure 4A). As illustrated in Figure 4B, Core 2 and ATP synthase α proteins were detected in the mitochondrial fraction, whereas only traces were present in the cytosolic fraction. Similar observations were done for the 13.4 kDa subunit of complex III (data not shown) and citrate synthase (Figure 4C). Altogether, these data indicate that the reported increased in mitochondrial protein expression was not associated with a defect in mitochondrial protein import and that the decrease in mitochondrial respiration observed in R1H cells was rather due to a functional deficit in one or several complexes of the respiratory chain.

### Mitochondrial H_2_O_2_ Level

**Figure 5:**
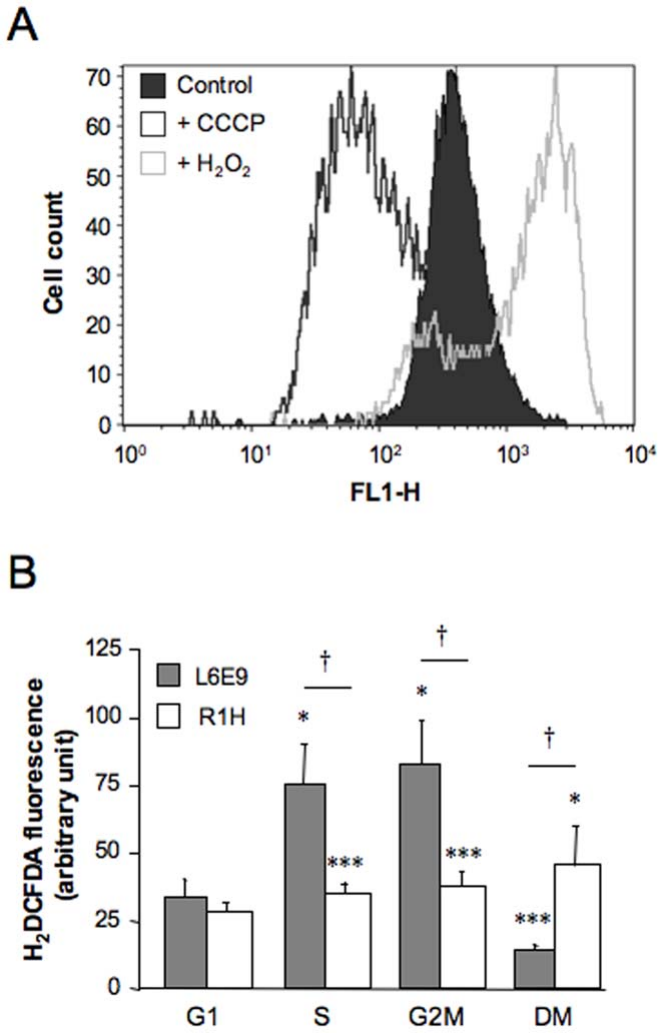
Mitochondrial H_2_O_2_ level. **(A)** Representative frequency histogram of 2′,7′-dichlorodihydrofluorescein diacetate (H_2_DCFDA) fluorescence (FL1-H) obtained in L6E9 myoblasts cultured in growth medium. Positive and negative controls were obtained by incubating cells with 100 µM H_2_O_2_ (right displacement) and 1 mM CCCP (left displacement), respectively. (B) H_2_DCFDA fluorescence was determined from the geometric means of fluorescence intensity peaks. Analyses were performed on morphologically normal living cells cultured in growth medium (G1, S and G2M phases) and differentiation medium (DM) for 36 hours. Data are means ± SE from 6 culture dishes. * *P*<0.05 and *** *P*<0.001: significantly different from corresponding cells in G1 phase; † *P*<0.05: significantly different from L6E9 myoblasts.

We next addressed the biological relevance of the reported deficiency in mitochondrial respiratory capacity of R1H cells. As an organelle participating in cell signaling [27], we hypothesized that the mitochondrial deficiency reported herein could alter the production of signaling agents involved in the regulation of myogenic differentiation. We particularly investigated H_2_O_2_ production as mitochondria are a major site of free radical production in the cell and this production is frequently altered in tumor tissues [28]. Furthermore, an increase in H_2_O_2_ concentration has been shown to completely abolish the differentiation of myoblasts into myotubes [29] and the stimulation of mitochondrial ROS production induced a rhabdomyosarcoma-like phenotype in human fibroblasts [14]. In the present study, mitochondria were identified as a major source of H_2_O_2_ as illustrated by a marked reduction in fluorescence of H_2_DCFDA, a marker of mitochondrial H_2_O_2_ level [30], after the addition of the mitochondrial uncoupler CCCP (Figure 5A). Striking differences were observed in the regulation of mitochondrial H_2_O_2_ production. The two-fold increase in H_2_DCFDA fluorescence during the transition from G1 to S phases in L6E9 cells was not observed in R1H cells (Figure 5B). Most importantly, the strong reduction in mitochondrial H_2_O_2_ level that occurred when cells were switched to a differentiation medium did not occur in R1H cells. We therefore tested the possibility that an antioxidant supplementation would force R1H cells to exit the cell cycle. Preincubation of the cells with 20 mM N-acetyl cysteine, which was effective in reducing basal H_2_O_2_ production, did not induce cell cycle arrest of R1H cells (data not shown).

## Discussion

Rhabdomyosarcoma arises as a consequence of an unbalance between proliferation and differentiation of myoblasts that maintains myoblasts in replicative state. Rhabdomyosarcoma cells thus constitute an interesting model to study the cellular mechanisms that control the myogenic differentiation program. The present study supports the hypothesis that a deficiency in mitochondrial metabolism is an epigenetic mechanism that contributes to the failure of rhabdomyosarcoma cells to differentiate. Corollary, it also supports the notion that a tight regulation of mitochondrial biogenesis and mitochondrial metabolism is necessary for the proper engagement of myoblasts towards the myogenic fate.

The composition of proteins and phospholipids of the mitochondrial inner membrane is crucial for mitochondrial function. Here, we show that a progressive depletion of the cardiolipin content occurs as R1H cells divide, while mitochondrial content is preserved. The biogenesis of cardiolipin and the relative distribution of the major lipid components of mitochondria (cardiolipin, phosphatidylcholine, phosphatidylethanolamine) are therefore profoundly modified in R1H cells. Alteration in organelle biogenesis was also observed at the protein level, since expression of mitochondrial respiratory chain proteins (core II, 13.4 and 20D6) and mitochondrial biogenesis factors (PGC-1α, PPARα, PPARδ and NOS1) was significantly increased. Specifically, the repression of mitochondrial proteins and mitochondrial biogenesis factors expression that occurs in L6E9 cells at the onset of differentiation (present study, [25]), did not occur in R1H cells. Taken together, these data clearly illustrate that the regulation of mitochondrial biogenesis is strongly altered in R1H cells.

The dysregulation of mitochondrial biogenesis observed in R1H cells was associated with a major alteration in mitochondrial metabolism. Oxygen consumption dedicated to drive ATP synthesis and mitochondrial respiratory reserve were dramatically reduced in R1H cells, leading mitochondria of R1H cells to be almost maximally stimulated under basal respiration. R1H cells seem to compensate the decrease in mitochondrial metabolism by increasing ATP production through lactate production. However, this metabolic pathway may not be sufficient to produce enough ATP when a large energy demand is required [15], as occurring during myoblast proliferation and differentiation. Therefore, such bioenergetic features suggest that R1H cells have a drastically lower capacity to face a metabolic aerobic challenge, despite the higher rate of lactate production. From a teleological point of view, the marked increase in mitochondrial protein expression and mitochondrial biogenesis factors reported therein may be interpreted as a vain attempt of R1H cells to counteract the decrease in mitochondrial respiratory capacity, as previously observed in mitochondrial myopathies [31] and in response to mitochondrial DNA depletion [32]. To explain the paradoxical observation that an increased mitochondrial protein content was not translated into functional respiratory capacity in R1H cells, we hypothesized that a defect in mitochondrial protein import could lead to a cytosolic accumulation of mitochondrial proteins. However, this was not the case as our cellular fractionation analysis clearly indicates that mitochondrial respiratory proteins encoded by the nuclear genome (Core 2 and 13.4 kDa subunits of complex III, ATP synthase subunit α and citrate synthase) are effectively imported inside the mitochondria. Another possibility would be that the observed changes in the protein-to-cardiolipin ratio alter the functionality of the respiratory chain and/or the assembly of respiratory complexes in R1H cells. In agreement with this hypothesis, previous studies have demonstrated that experimental (mutant deficient in cardiolipin) and physiological (aging) conditions leading to cardiolipin depletion, markedly decrease the respiratory capacity of mitochondria [33], [34]. Importantly, of the mitochondrial respiratory chain complexes, complex I was the most severely impaired [34]. Together with the observation that complex I activity is reduced in R1H cells [35], our report that cardiolipin content is strongly decreased in R1H cells may thus contribute to explain the decrease in the respiratory capacity of R1H cells. Overall, these data suggest that a tight regulation between protein and phospholipid syntheses is necessary to produce functional mitochondria.

H_2_O_2_, the intermediate detoxification product of superoxide ion by superoxide dismutase, is involved in the regulation of myogenic differentiation. It has been notably reported that exposure of differentiating myoblasts to H_2_O_2_ almost totally abolished muscle-specific protein expression and myogenic differentiation, whereas this effect was reversed by the addition of a reactive oxygen species scavenger [29]. A decrease in H_2_O_2_ level at the onset of differentiation would be therefore a permissive event necessary for the continuation of the myogenic program. Importantly, ion superoxide production and the subsequent production of H_2_O_2_ are tightly coupled to the activity of complexes I and III of the respiratory chain [36], [37], and the cardiolipin content of the inner mitochondrial membrane [33]. Therefore, the reported deficiency in mitochondrial respiration and the decrease in cardiolipin content of R1H cells may profoundly impact the regulation of mitochondrial H_2_O_2_ production and ultimately contribute to the failure of R1H cells to differentiate. In agreement with this hypothesis, H2O2 production was fairly constant in R1H cells induced to differentiate, which contrasted with the decrease in H2O2 level occurring at the onset of differentiation in L6E9 myoblasts. However, supplementation of R1H cells with N-acetyl cysteine, a powerful antioxidant molecule, did not rescue the failure of R1H cells to differentiate, suggesting that other factors must contribute to the failure of R1H cells to differentiate.

In summary, we identified a mitochondrial respiratory deficiency in R1H rhabdomyosarcoma cells that may result from an unbalance between mitochondrial protein expression and cardiolipin biosynthesis. A defect in the regulation of mitochondrial biogenesis and mitochondrial metabolism may thus be an epigenetic mechanism that may contribute to the tumoral behavior of R1H cells. Furthermore, our data further establish functional links between the regulation of mitochondrial biogenesis and the regulation of myogenic differentiation.

## Materials and Methods

### Cell Culture

Experiments were performed on rat L6E9 muscle cells (gift from Dr D.A. Hood, York University, Canada) and rat rhabdomyosarcoma tumor cells (gift from Dr A. Raabe, University Medical Center Hamburg-Eppendorf, Germany). The tumor was derived from the rhabdomyosarcoma R1H [38], which was originally derived from the BA1112 tumor [39]. Cells were cultured in Dulbecco's modified Eagle's medium (DMEM) supplemented with 20% fetal bovine serum and 1% penicillin-streptomycin (P/S) at 37°C and 5% CO_2_ in air, in 100 mm plastic dishes. At 80% confluence, cells were either trypsinized or allowed to differentiate for 36 hours in DMEM supplemented with 2% horse serum and 1% P/S.

### Flow Cytometry Analyses

Cells (10^6^ cells/ml) were analyzed on a FACSDiva (BD Biosciences, San Jose, CA, USA). Before analyses, cells were gated to perform measurements on morphologically normal single living cells (doublet exclusion and propidium iodide staining) as a function of the position of cells in G1, S, and G2M phases of the cell cycle (9 µM Hoechst 33342) [25]. Cell cycle was analyzed with ModFit™ 3.1 software. Data were analyzed using BD Diva™ 5.0.3 or BD Cell Quest Pro™4.0.2.

Cardiolipin content, mitochondrial content, and mitochondrial H_2_O_2_ level. Nonyl acridine orange (NAO, Sigma), Mitotracker® Deep Red FM (Invitrogen) and 2′,7′-dichlorodihydrofluorescein diacetate (H_2_DCFDA, Invitrogen) were used to determine cardiolipin content [24], mitochondrial content [40] and mitochondrial H_2_O_2_ level [30], respectively. NAO and H_2_DCFDA stainings were performed according to [25] with some modifications. NAO and H_2_DCFDA concentrations were 2 µM for R1H cells. For Mitotracker® Deep Red FM staining, cells (10^6^/ml) were successively incubated at 37°C with Hoechst 33342 (9 µM, 90 min) and Mitotracker® Deep Red FM (1 µM, 15 min).

Immunolabelling experiments. Cell immunoreactivity against complex III (Core II and 13.4 subunits) and ATP synthase α (20D6) of the respiratory chain, proliferating cell nuclear antigen (PCNA, PC10, NeoMarkers), peroxysome proliferator activated receptor (PPAR) γ co-activator-1α (PGC-1α, sc-13067, Santa Cruz Biotechnology), PPARα (sc-9000, Santa Cruz Biotechnology), PPARδ (sc-7197, Santa Cruz Biotechnology) and neuronal nitric oxide synthase (NOS1, sc-648, Santa Cruz Biotechnology) were performed as previously described [25].

### Fluorescence Microscopy

Myoblasts and rhabdomyosarcoma cells were cultured on coverslips in DMEM supplemented with 20% fetal bovine serum and 1% P/S. Cells were stained with NAO as described [25]. Fluorescence was visualized with an Olympus inverse fluorescent microscope IX81 system.

### Mitochondrial DNA Analysis

Total DNA was extracted from muscle cells using phenol/chloroform/isoamyl alcohol (25∶24∶1) followed by ethanol precipitation. The content of mtDNA was calculated using real-time quantitative PCR by measuring the threshold cycle ratio (ΔCt) of a mitochondrial-encoded gene (subunit II of cytochrome oxidase, forward 5′-CTTACAAGACGCCACATCAC-3′, reverse 5′-GAATTCGTAGGGAGGGAAGG-3′) versus a nuclear-encoded gene (peptidylprolyl isomerase A, forward 52-ACACGCCATAATGGCACTGG-32, reverse 52-CAGTCTTGGCAGTGCAGAT-32). mtDNA-to-nuclear DNA ratio were normalized to the protein content.

### Cell Respiration

L6E9 and R1H cells were trypsinized, washed and finally suspended at a final concentration of 2×10^6^ cells per ml in serum-free DMEM. Cell respiration was measured as previously described [15]. Briefly, the cell suspension (500 µl) was immediately transferred to a 1 ml chamber of a Clarke-type oxygen electrode (Hansatech, UK) maintained at 37°C using a recirculating water bath. Basal cell respiration is the sum of the oxygen consumed for mitochondrial ATP synthesis, mitochondrial proton leak and non-mitochondrial reactions. Myxothiazol (8 µM) was used to check potential non-mitochondrial sources of oxygen consumption. Non-mitochondrial respiration rates were considered insignificant as myxothiazol-inhibited respiration was not detectable. Basal endogenous coupled respiration rate of cells was determined by measuring the linear rate of oxygen consumption. Oligomycin (10 µg/ml) was then added to inhibit ATP synthase (non-phosphorylating respiration). Carbonyl cyanide-p-trifluoromethoxyphenylhydrazone (FCCP) was sequentially added at different concentrations ranging from 500 nM to 900 nM. This titration was systematically performed in order to determine the optimal FCCP concentration that gives maximal uncoupled respiration rate. Using this approach, mitochondrial proton leak (oligomycin-insensitive respiration rate), mitochondrial ATP synthesis (basal respiration rate minus oligomycin-insensitive respiration rate) and mitochondrial respiratory reserve (FCCP-induced maximal respiration rate minus basal respiration rate) were determined. Oxygen calibration was performed by adding Na_2_S_2_O_4_.

### Lactate Measurement

L6E9 and R1H cells were cultured as described above in 100 mm plastic dishes. At the end of the culture period (72 hours for cells in proliferation and 36 hours for cells in differentiation medium), cells were rinsed, trypsined and numbered. Lactate concentrations were determined on culture media obtained at the beginning and the end of the culture period by using an electrochemical analyser YSI 2300 STAT PLUS (Yellow Springs Instruments, Yellow Springs , Ohio, USA). Calibration was done by using a 5 mM standard lactate solution.

### Core 2 Subunit of Complex III and ATP Synthase α Protein Content in Cytosolic and Mitochondrial Fractions

Mitochondrial and cytosolic fractions from L6E9 and R1H cells were prepared using the MITOISO2 kit (Sigma) following the manufacturer's instructions. Purity of mitochondrial and cytosolic fractions was systematically assessed by the measurement of lactate dehydrogenase enzyme activities according to [41]. Equal amount of cells (100,000 and 200,000 cells) were then separated on a 12.5% SDS-PAGE. After electrotransfer, the membranes were saturated with 5% (w∶v) non-fat dry milk in Tris-buffered saline (1 hour at room temperature) and then incubated overnight with the primary antibody either directed against the Core 2 subunit of complex III (13.4) (1∶10,000 dilution v∶v) or the ATP synthase α (20D6) (1∶40,000 v∶v). Corresponding secondary antibodies (1∶3,000 dilution v∶v) were incubated for 90 min. Protein immunoreactivity was determined by chemiluminescence. Quantification of the signal intensity was determined on scanned films by using NIH image 1.63. Finally, citrate synthase activity was also determined on cytosolic and mitochondrial fractions [41].

### Supplementation with N-Acetyl Cysteine

R1H cells were supplemented with 20 mM N-acetyl cysteine (Sigma) in the culture medium over the entire differentiation period (36 hours). Cells were then trypsinized and stained with 9 µM Hoescht 33342 for a cell cycle analysis [25].

### Statistics

Data are means ± SE from 6 independent culture dishes. Mean difference between myoblasts and rhabdomyosarcoma cells in G1, S and G2M was determined by using a two-way analysis of variance. Scheffe post-hoc test was used to identify specific mean difference. Unpaired *t* test was used to determine specific mean differences between myoblasts cultured in growth and in differentiation media. Unpaired *t* test was also used to determine mean difference between myoblasts in G1 and at the onset of differentiation. The α level of significance was set at 0.05.
